# Biogeographic distribution of five Antarctic cyanobacteria using large-scale k-mer searching with sourmash branchwater

**DOI:** 10.3389/fmicb.2024.1328083

**Published:** 2024-02-19

**Authors:** Jessica Lumian, Dawn Y. Sumner, Christen L. Grettenberger, Anne D. Jungblut, Luiz Irber, N. Tessa Pierce-Ward, C. Titus Brown

**Affiliations:** ^1^Department of Earth and Planetary Sciences, Microbiology Graduate Group, University of California Davis, Davis, CA, United States; ^2^Department of Earth and Planetary Sciences, University of California Davis, Davis, CA, United States; ^3^Department of Environmental Toxicology, University of California Davis, Davis, CA, United States; ^4^Department of Science, The Natural History Museum, London, United Kingdom; ^5^Population Health and Reproduction, University of California Davis, Davis, CA, United States

**Keywords:** biogeography, bioinformatics, cyrosphere, polar cyanobacteria, metagenomics

## Abstract

Cyanobacteria form diverse communities and are important primary producers in Antarctic freshwater environments, but their geographic distribution patterns in Antarctica and globally are still unresolved. There are however few genomes of cultured cyanobacteria from Antarctica available and therefore metagenome-assembled genomes (MAGs) from Antarctic cyanobacteria microbial mats provide an opportunity to explore distribution of uncultured taxa. These MAGs also allow comparison with metagenomes of cyanobacteria enriched communities from a range of habitats, geographic locations, and climates. However, most MAGs do not contain 16S rRNA gene sequences, making a 16S rRNA gene-based biogeography comparison difficult. An alternative technique is to use large-scale k-mer searching to find genomes of interest in public metagenomes. This paper presents the results of k-mer based searches for 5 Antarctic cyanobacteria MAGs from Lake Fryxell and Lake Vanda, assigned the names *Phormidium pseudopriestleyi* FRX01, *Microcoleus* sp. MP8IB2.171, *Leptolyngbya* sp. BulkMat.35, *Pseudanabaenaceae cyanobacterium* MP8IB2.15, and *Leptolyngbyaceae cyanobacterium* MP9P1.79 in 498,942 unassembled metagenomes from the National Center for Biotechnology Information (NCBI) Sequence Read Archive (SRA). The *Microcoleus* sp. MP8IB2.171 MAG was found in a wide variety of environments, the *P. pseudopriestleyi* MAG was found in environments with challenging conditions, the *Leptolyngbyaceae cyanobacterium* MP9P1.79 MAG was only found in Antarctica, and the *Leptolyngbya* sp. BulkMat.35 and *Pseudanabaenaceae cyanobacterium* MP8IB2.15 MAGs were found in Antarctic and other cold environments. The findings based on metagenome matches and global comparisons suggest that these Antarctic cyanobacteria have distinct distribution patterns ranging from locally restricted to global distribution across the cold biosphere and other climatic zones.

## 1 Introduction

Cyanobacteria are a diverse group of oxygenic photosynthetic bacteria that are prevalent in a wide range of environments. In terrestrial polar environments, such as lakes, ephemeral streams, and soils, cyanobacteria play an important part in supporting local ecosystems because of their role as primary producers (Stal, 2007; Quesada and Vincent, 2012; Chrismas et al., 2016). Cyanobacteria that thrive in Antarctica face many challenges including variable light availability, cold temperatures, and freeze-drying conditions. To withstand these conditions, cyanobacteria may have tolerance mechanisms encoded in their genomes (Chrismas et al., 2015, 2016). However, the presence of tolerance genes in their genomes may make it more difficult for polar cyanobacteria to compete with other cyanobacteria in non-polar environments. Consequently, some polar cyanobacteria may only occur in the extremes of polar environments, while others may also be present in environments that share similar conditions to the stresses they face in Antarctica, such as cold temperatures or light stress (Jungblut et al., 2016; Chrismas et al., 2018; Lumian et al., 2021).

Currently, polar cyanobacteria are underrepresented in genomic databases, despite the important role they play in primary productivity. Recent research has focused on characterizing cyanobacteria in benthic biofilms in perennially ice-covered lakes in the McMurdo Dry Valleys in Antarctica (Sumner et al., 2015; Zhang et al., 2015; Jungblut et al., 2016; Dillon et al., 2020; Grettenberger et al., 2020; Lumian et al., 2021). Due to a lack of grazers and limited water mixing in these lakes, vast microbial mats prosper and sustain complex geochemical gradients (Jungblut et al., 2016; Sumner et al., 2016; Lumian et al., 2021). These geochemical gradients structure competition within communities, which are also dealing with challenging environmental conditions, such as highly seasonal irradiance, nutrient limitation, cold temperatures, and in some cases sulfidic water (Jungblut et al., 2016; Dillon et al., 2020; Lumian et al., 2021).

The question of why Antarctic cyanobacteria can survive in challenging conditions and what other environments they grow in can be addressed by biogeography studies (Whitaker et al., 2003; Martiny et al., 2006; Fierer, 2008; Green et al., 2008). Previous 16S rRNA gene surveys based on amplicon sequencing provided support for the longstanding theory that microbes have unlimited dispersal and community distribution is selected by the environment (Baas-Becking, 1934; Jungblut et al., 2010; Harding et al., 2011). However, studies from other environments and climatic zones have shown that 16S rRNA gene and single gene markers might not provide sufficient information to resolve genotypes and populations. Yet, most biogeography studies on polar microbiomes and cyanobacteria to date are based on 16S rRNA gene amplicon sequencing in the context of local environmental conditions of sampling sites or pole-to-pole comparisons using clone library surveys and high throughput sequencing approaches (Taton et al., 2006; Namsaraev et al., 2010; Bahl et al., 2011; Jungblut et al., 2010; Moreira et al., 2013; Harke et al., 2016; Kleinteich et al., 2017; Ribeiro et al., 2018). Although 16S rRNA gene sequences are computationally easier to compare to each other, there are limitations to 16S rRNA gene-based biogeography studies. The 16S rRNA gene is conserved and therefore likely leads to an under estimation of genotype level richness. Furthermore, the short read length of high throughput sequencing only allows the coverage of a few variable regions which further reduces phylogenetic resolution. While recent genomic work has provided advances in biogeography of polar microbes (Chrismas et al., 2015), the 16S rRNA gene sequence may not assemble and bin well from metagenomes, which can prohibit MAGs from being incorporated into 16S rRNA gene-based biogeographical distributions.

An alternative to 16S rRNA gene-based biogeography is to apply comparative genomic approaches, but this is computationally more complicated because of the size and scale of metagenome datasets. One option is to use an alignment-based approach in which the reads are aligned to reference genomes, which has been done for large-scale viral genome discovery with Serratus (Edgar et al., 2022). Another option is to apply large-scale k-mer matching to unassembled metagenomes, which can be implemented using software like sourmash and its specialized implementation branchwater (Brown and Irber, 2016; Irber, 2020a,b; Brown, 2021; Irber et al., 2022a,b). These techniques open the possibility of using metagenomic data for biogeography studies by searching all publicly available metagenomes on the National Center for Biotechnology Information (NCBI) Sequence Read Archive (SRA) (Leinonen et al., 2011) for MAGs of interest. In this paper, branchwater was used to search 498,942 unassembled metagenomes from the NCBI SRA for the presence of five Antarctic cyanobacteria MAGs that lack the 16S rRNA gene. Identifying global matches in the metagenomes allowed widespread biogeographical analyses. These findings provide new insights based on comparative genomic analyses into the distribution patterns of cyanobacteria in cold biospheres: some cyanobacteria MAGs were only found in cold or polar regions, while others were found in a variety of environmental conditions.

## 2 Materials and methods

### 2.1 Selection of Antarctic cyanobacteria

*Phormidium pseudopriestleyi* FRX01 is a well characterized cyanobacteria in Lake Fryxell, Antarctica (Lumian et al., 2021). Lake Fryxell is a perennially ice-covered lake located at 77.36° S, 162.6° E in the McMurdo Dry Valleys. The lake floor is covered with microbial mats to depths of almost 10 m, with *P. pseudopriestleyi* FRX01 dominating the mats at 9.8 m in depth in 2012, where light levels are low (1–2 μmol photons m-2 s-1) and sulfide is present in the water column (0.091 mg L^−1^) (Lumian et al., 2021). *P. pseudopriestleyi* FRX01 performs oxygenic photosynthesis in the presence of hydrogen sulfide, even though sulfide inhibits oxygenic photosynthesis (Sumner et al., 2015; Lumian et al., 2021). Lake conditions and sampling have been described in Jungblut et al. (2016), Dillon et al. (2020), and Lumian et al. (2021).

The *Leptolyngbyaceae cyanobacterium* MP9P1.79, *Leptolyngbya* sp. BulkMat.35, *Microcoleus* sp. MP8IB2.171, and *Pseudanabaenaceae cyanobacterium* MP8IB2.15 MAGs are from microbial mats sampled from Lake Vanda, McMurdo Dry Valleys. Lake Vanda is also a perennially ice-covered lake and is located at 77.53° S, 161.58° E. Microbial mats in Lake Vanda contain pinnacles that range from millimeters to centimeters tall. Unlike Lake Fryxell, there is no sulfide where we sampled, and it is better illuminated at the sampled location than Lake Fryxell, though samples from the inside of pinnacles receive little light (Sumner et al., 2016). Sampling methods and lake conditions have previously been described in Sumner et al. (2016).

### 2.2 Bioinformatic processing and assembly of Antarctic cyanobacteria reference MAGs

The methods to obtain the *P. pseudopriestleyi* FRX01 MAG (ASM1731333v1) has been previously described in Lumian et al. (2021). Briefly, the *P. pseudopriestleyi* FRX01 MAG was obtained from a microbial mat sample sequenced on an Illumina HiSeq 2,500 PE250 platform and a laboratory culture was sequenced on an Illumina 2000 PE100 platform. The microbial mat sample was quality filtered, and forward and reverse reads were joined using PEAR v0.9.6 (Zhang et al., 2014). For the isolated strain, trimmomatic v0.36 (Bolger et al., 2014) was used to trim sequencing adapters, and the interleave-reads.py script in khmer v2.1.2 (Crusoe et al., 2015) was used to interleave the reads. Both samples were assembled separately and together as a co-assembly by MEGAHIT v1.1.2 (Li et al., 2015) and mapped with bwa v2.3 (Li, 2013) and samtools v1.9 (Li et al., 2009). A single cyanobacteria bin was obtained using the CONCOCT binning algorithm in anvi’o and identified using CheckM (Eren et al., 2015; Parks et al., 2015; Delmont and Eren, 2018). The *P. pseudopriestleyi* FRX01 bin was refined with spacegraphcats to extract additional content from the metagenomes with a k-mer size of 21 and a radius of 1 (Brown et al., 2020).

Methods to obtain the *Microcoleus* sp. MP8IB2.171, *P. cyanobacterium* MP8IB2.15, *Leptolyngbya* sp. BulkMat.35, and *Leptolyngbyaceae cyanobacterium* MP9P1.79 MAGs from Lake Vanda were described in (Lumian et al., 2024). Filtered and quality controlled raw data was retrieved from the NCBI Sequence Read Archive under the accession numbers SRR6448204 - SRR6448219 and SRR 6831528. MEGAHIT v1.9.6 was used to assemble metagenomes with a minimum contig length of 1,500 bp and a paired end setting. Bowtie2 v1.2.2 and samtools v1.7 were used to map reads back to the assembly. A depth file was generated using jgi_summarize_bam_contig_depths from MetaBAT v2.12.1 (Kang et al., 2015), which was also used to generate bins with a minimum contig length of 2,500 bp. The completeness and contamination of the bins were calculated with CheckM v1.0.7 (Parks, D.H., et al., 2014). Bins that were contained within the phylum Cyanobacteriota in the phylogenetic tree generated by CheckM were retained for further analysis. 139 single copy marker genes (Campbell et al., 2013) were collected using the anvi-run-hmms command in anvi’o v6.2 (Eren et al., 2021) and a phylogenetic tree was constructed using the anvi-gen-phylogenomic-tree command. Genome similarity between bins was computed using the anvi-compute-genome-similarity command. Bins were grouped into taxa if they shared more than 98% average nucleotide similarity and were phylogenetically cohesive. When a taxon was found in multiple metagenomes, the most complete bin with the lowest level of contamination for that taxon was selected for additional analysis and was referred to as the MAG for that taxon. Taxa were classified using GTDB-tk v.2.1.0 (Chaumeil et al., 2020). MAGs for each taxon are available in the NCBI sequence read archive under the accession numbers: ASM1731333v1, JARCMA000000000.1, JARCMB000000000.1, JARCMC000000000.1, JARCMD000000000.1.

### 2.3 Sourmash branchwater software with large-scale k-mer searching for comparative metagenomic analysis

The branchwater software used large-scale k-mer searching to search metagenomes in the NCBI SRA for matches with genomes of interest (Brown and Irber, 2016; Pierce et al., 2019). Signature files of the genomes of interest were generated using sourmash v3.5.0 (Brown and Irber, 2016) with k-mer sizes of 21, 31, 51, the scaled parameter set to 1,000, and abundance tracking. This generated a unique signature file specific to each of the five Antarctic MAGs. These signature files were searched against signature files previously generated for 498,942 publicly available unassembled metagenome sets on the September 2020 branchwater SRA database using exact k-mer matching. Results are organized by containment, which is the proportion of the query MAG k-mers found in the metagenome. Branchwater also provides Average Nucleotide Identity (ANI) values estimated from k-mer containment; the use of k = 31 as a k-mer size enables detection of matches to ~91% ANI at 5% containment and ~ 96% ANI at 30% containment (Irber et al., 2022a,b; Hera et al., 2023). The size of the Antarctic query MAGs ranged from 2.7 Mbp – 6.07 Mbp, so a match with containment value of 5% implies 135,000–303,500 matching k-mers with k = 31 and 4,185,000 – 9,408,500 matching base pairs, which indicates significant shared genomic material between MAGs and metagenome matches. The number of matching bases pairs also depends on the depth of metagenomic sequencing and sample community characteristics, including the ANI similarity of organisms to the query MAG, their abundance in the community, and the diversity of the community. Thus, low containment does not demonstrate the absence of an organism. However, high containment requires ANI similarity of an organism that has sufficient abundance to have its genome content well represented in the metagenome.

Validation of k-mer results from branchwater was done by mapping the Antarctic MAGs back to the metagenomes from the SRA using minimap2 v2.24 in genome-grist v0.8.4 (Li, 2018; Irber et al., 2022a,b). Environmental metadata for the top hits of all MAGs with hits above 5% were recorded, except for *Microcoleus* sp. MP8IB2.171, which had over 1,000 matches above that threshold (Table 1; Supplementary Tables S1, S2).

**Table 1:** Summary of branchwater hits.

	*Microcoleus* sp. MP8IB2.171	*P. pseudopriestleyi* FRX01	Pseudanabaenaceae cyanobacterium MP8IB2.15	Leptolyngbyaceae cyanobacterium MP9P1.79	*Leptolyngbya* sp. BulkMat.35
# Hits >75% Containment	6	30	6	3	5
# Hits >50% Containment	12	33	6	5	5
# Hits >25% Containment	119	38	10	6	6
# Hits >5% Containment	1,121	131	24	16	22
Total Hits	6,184	2,739	3,769	2,796	2,999
# Geographically Distinct Locations >25% Containment	27	3	3	1	1

Environments with >30% containment are marked for *Microcoleus* sp. MP8IB2.171. Environment markers in the McMurdo Dry Valley are shown next to each other instead of stacked for clarity. World map base image is from Wikimedia Commons.

In an effort to generate MAGs of our five taxa of interest in metagenomes with >5% containment, we retrieved the relevant unassembled metagenomes. Unassembled metagenomes from geographically distinct environments were assembled with MEGAHIT v1.9.6, mapped with bowtie2 v1.2.2 and samtools v1.7, and binned with MetaBAT v.2.12.1. However, none of the assemblies were high enough quality to yield bins (Supplementary Table S3). The code from this project is available at: https://github.com/dib-lab/2022-pipeline-antarctic-biogeography

## 3 Results

**Figure 1:**
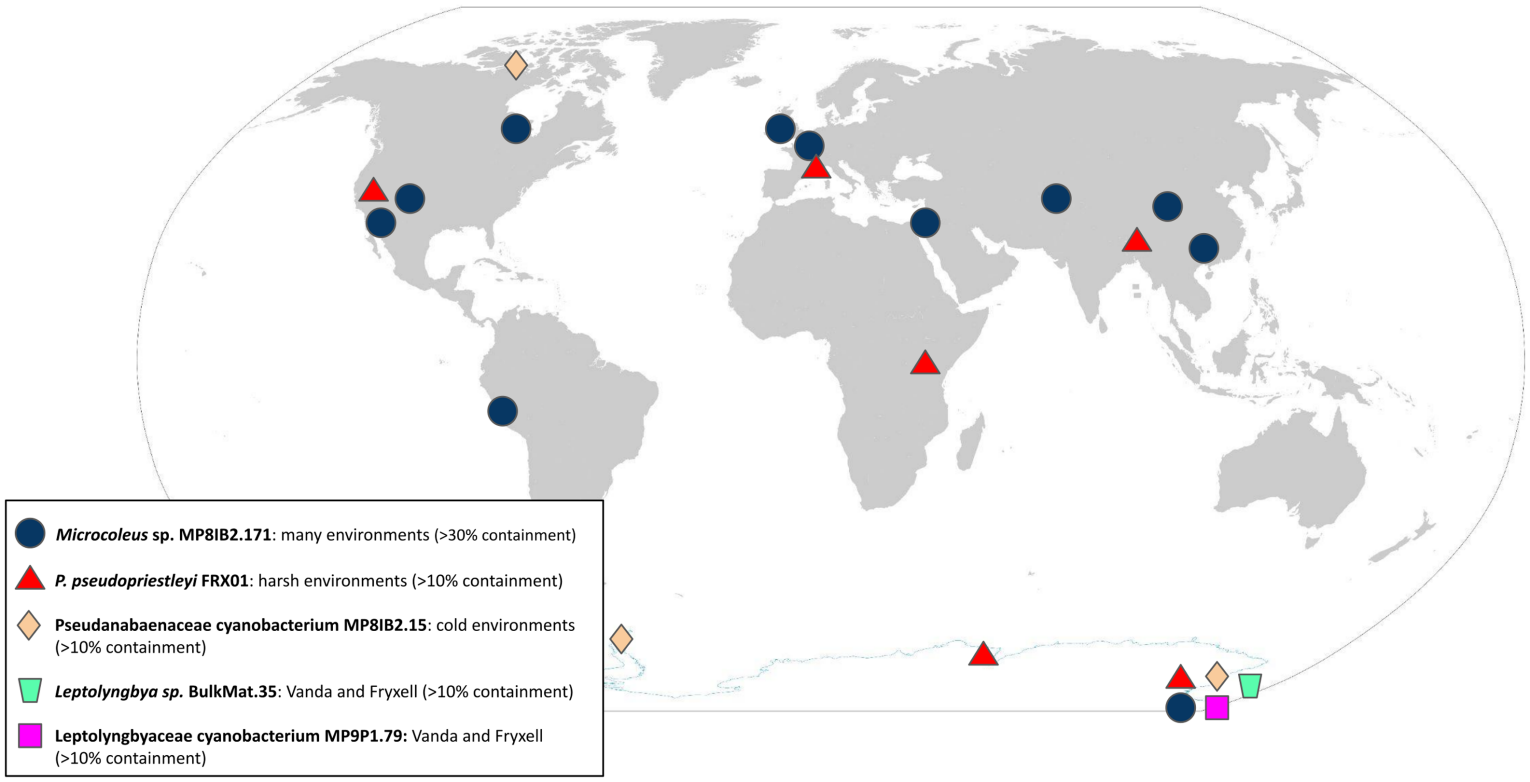
Global distribution of query MAGs. Environments with >10% containment are marked for *P. pseudopriestleyi* FRX01, Pseudoanabaenaceae cyanobacterium MP8IB2.15, and *Leptolyngbya* sp. BulkMat.35. (File:WorldMap.svg, 2022).

The five polar cyanobacteria MAGs used as search queries were found in a variety of non-polar metagenomic data sets in a range of environmental conditions (Table 1; Figure 1). The metagenome data sets with >5% containment of the MAGs described in Supplementary Tables S1, S2. Information about additional environments where the *Microcoleus* sp. MP8IB2.171 MAG was found with over 20% containment is displayed in Supplementary Table S2. Validation mapping data are available in Table 2. The SRA accession numbers of additional hits are available in Supplementary Tables S4–S8.

**Table 2:** Mapping validation of MAGs in SRA metagenomes.

MAG	SRA accession number and location	K-mer containment (%)	Effective coverage	Percentage of MAG detected in metagenome (%)	Number of mapped reads from MAG
*Microcoleus* sp. MP8IB2.171	SRR5468150 Mat lift-off from Lake Fryxell, Antarctica	99.18^*^	125.84	99.35	5,864,248
	SRR6266358 Polar Desert Sand Communities, Antarctica	65.02^*^	93.34	88.34	3,832,909
	SRR5855414 Moab Green Butte, Utah, USA	57.50^*^	407.19	86.11	15,915,624
	SRR2952554 Ningxia, China	41.65^*^	18.83	73.53	899,792
	SRR5247052 Sonoran Desert, Colorado Plateau, USA	41.10^*^	180.87	73.08	10,101,904
	ERR3588763 Pig Farm, UK	40.61^*^	9.38	76.14	329,215
	SRR5891573 Glacier Snow, China	39.54^*^	14.36	75.66	482,590
	ERR1333181 Mine Tailing Pool Sediment near Shaoyang, China	38.36^*^	28.59	73.24	1,120,980
	SRR5459769 Wastewater in Milwaukee, Wisconsin, USA	37.04^*^	13.67	76.29	636,988
	SRR6048908 Puca Glacier, Peru	36.30^*^	7.76	73.49	280,909
	SRR12473531 Negev Desert, Israel	35.71^*^	18.06	74.46	639,468
	ERR3192241 Southwest Germany	33.58^*^	8.80	69.98	288,838
*P. pseudopriestleyi* FRX01	SRR7769747 Microbial mat in Lake Fryxell	98.49^*^	22.61	98.93	602,728
	SRR7528444 Ace Lake, Antarctica	55.80^*^	2.21	61.50	103,088
	SRR5216658 Rauer Islands, Antarctica	23.54^*^	1.59	27.23	21,770
	SRR7428116 Les Salins du Lion Bird Reserve, France	20.63*	11.68	60.33	471,918
	SRR12522841 Big Soda Lake, Nevada	19.04^*^	10.67	67.52	340,050
	SRR7428132 Étang de Berre Lagoon, France	18.25^*^	2.75	47.58	90,501
	ERR3503286 Sewage in Nairobi, Kenya	11.99^*^	2.12	40.00	38,112
	SRR9691033 Wetland soil in Yanghu, China	10.37	1.69	30.33	24,130
	SRR10186387 Salar del Huasco salt flat, Chile	8.98^*^	3.64	24.24	50,222
	ERR738546 Simulated Metagenome	8.48^*^	1.45	19.54	20,152
	SRR6262267 Human Gut	7.61^*^	2.06	25.27	25,271
P. cyanobacterium MP8IB2.15	SRR5468149 Mat lift-off from Lake Fryxell, Antarctica	99.49*	142.44	99.89	2,748,583
	SRR6266338 Dry Valley Sand Communities, Antarctica	37.45	2.65	45.28	28,689
	SRR5829599 Nunavut, Canada	33.54^*^	5.91	78.56	97,210
	ERR4192538 Deception Island, Antarctica (Whaler’s Bay Sediment)	18.31^*^	2.48	46.67	24,011
	SRR7769784 Microbial mat in Lake Fryxell	7.45^*^	1.13	9.00	1,405
L. cyanobacterium MP9P1.79	SRR5208701 Mat lift-off from Lake Fryxell, Antarctica	97.82^*^	4.023	90.79	133,786
*Leptolyngbya* sp. BulkMat.35	SRR5468150 Mat lift-off from Lake Fryxell, Antarctica	98.72^*^	62.71	99.35	2,759,236
	SRR6683740 Spitsbergen, Svalbard, Norway	8.40	1.71	16.67	17,394

The purpose of applying branchwater was to find shared genomic data between Antarctic MAGs and SRA metagenomes from different habitats, geographic locations, and climate zones. Matches of our selected Antarctic cyanobacteria MAGs in these metagenomes may indicate the occurrence of Antarctic cyanobacteria or closely related taxa in environments across the globe. A k-mer size of 31 with at least 5% containment indicates a ~ 91% ANI between matched sequences; at 30% containment, this value increases to ~96% ANI (Hera et al., 2023). Thus, a high containment value indicates the presence in the metagenome of genomic DNA similar to the MAG and supports the presence of a closely related organism in the sampling location of that metagenome. Low k-mer containment values may represent smaller regions of shared genomic material or the presence of a related species but cannot definitively support the presence of the same species in that environment. Containment, particularly at low values, can be affected by factors such as plasmids, low population size relative to metagenome sequencing depth, or small portions of shared contamination between the MAG and metagenome.

The number of metagenome samples with containment for the 5 MAGs depends both on the distribution of available metagenomes and on the distribution of the MAG within ecosystems. Only about 3% of the SRA metagenomes contained matches to any of the k-mers in the query MAGs. Many of the metagenomes available in the SRA were from dark environments that are not expected to support growth of cyanobacteria; only about 5,000 metagenomes contain “photic” within their metadata. Significantly more research needs to be done to understand how to interpret the proportion of total samples with containment for MAGs of different types of organisms.

Even though the larger context of the low proportion of metagenomes containing our MAGs is poorly constrained, variations in relative containment for the cyanobacteria represented by the 5 query MAGs are robust because all 5 were searched for in the same way across the same dataset. Interpretations of their geographic distribution must be contextualized relative to available metagenomes, which are biased by prior sampling. Biases in the SRA metagenome data set also raise questions concerning environmental interpretations. In many cases, the metadata associated with metagenomes do not provide sufficient environmental context (e.g., irradiance, pH, abundance of important nutrients, and other geochemical parameters) for robust comparisons among environments. In some cases, the environment of sampling appears inconsistent with cyanobacterial growth (e.g., infant gut; Supplementary Table S1), raising questions about the cause of the detection (e.g., Kennedy et al., 2023). We choose to include these environments in our discussion for completeness and moderate our interpretations of environmental context based on available data.

The *Microcoleus* sp. MP8IB2.171 MAG was the most widely distributed MAG with 27 globally distinct locations above 25% containment (Table 1). The *Microcoleus* sp. MP8IB2.171 and *P. pseudopriestleyi* FRX01 MAGs were present in the most time series and subsamples from the same environmental location, which resulted in 1,121 hits above 5% for the *Microcoleus* sp. MP8IB2.171 MAG and 131 hits for *P. pseudopriestleyi* FRX01 MAG (Table 1). The *Pseudanabaenaceae cyanobacterium* MP8IB2.15 and *P. pseudopriestleyi* FRX01 MAGs were found in three distinct locations above 25% containment while the *Leptolyngbyaceae cyanobacterium* MP9P1.79 and *Leptolyngbya* sp. BulkMat.35 MAGs were only found in one location each above 25% containment (Table 1).

The *Microcoleus* sp. MP8IB2.171 MAG was found in diverse environments with conditions ranging from hot to cold climates and including both arid and wet locations (Supplementary Tables S1, S2). Some environments are cold year-round such as Puca Glacier in Peru (36.30% containment), glacier snow in China (39.54% containment), and the ice-covered Lake Vanda, whereas others are temperate, like Wisconsin, USA (37.04% containment), or Southwest Germany (33.58% containment). *P. pseudopriestleyi* FRX01 MAG was found in three Antarctic metagenome data sets: Lake Fryxell mat samples (98.49% containment), Ace Lake (55.8% containment) and the Rauer Islands (23.54% containment). The highest 30 hits for the *P. pseudopriestleyi* FRX01 MAG, including the three samples used to create the MAG, were from Lake Fryxell. This search revealed that *P. pseudopriestleyi* FRX01 is likely present in other depths of Lake Fryxell than 9.8 m despite not being prevalent at those depths based on 16S sequencing (Jungblut et al., 2010). Besides Antarctica, the *P. pseudopriestleyi* FRX01 MAG was found in a bird reserve next to a lagoon in France called Les Salins du Lion (20.63% containment) as well as a hydrocarbon polluted saline lagoon called Étang de Berre (18.25% containment), which were part of a study on the effects of hydrocarbon pollution on microbial communities (Aubé et al., 2016). The *P. pseudopriestleyi* FRX01 MAG was also found in the Salar del Huasco salt flat in Chile (8.98% containment) and antimicrobial treated sewage collected in Nairobi, Kenya (11.99% containment). All these environments represent extreme conditions for cyanobacteria. This MAG was also found in an infant gut fecal sample (7.61% containment). This is likely due to contamination of the sample or from ingestion. However, non-photosynthetic Cyanobacteria (Vampirovibronia or Melainabacteria) are interpreted as living in human guts (e.g., Di Rienzi et al., 2013), and the relatively low containment might indicate the presence of an organism with genetic material shared with the *P. pseudopriestleyi* FRX01 MAG.

Although the *Microcoleus* sp. MP8IB2.171, *P. cyanobacterium* MP8IB2.15, *Leptolyngbyaceae cyanobacterium* MP9P1.79, and *Leptolyngbya* sp. BulkMat.35 MAGs were obtained from microbial mat pinnacles in Lake Vanda, they were all present in high containment (>97%) in mat lift-off samples from Lake Fryxell where the *P. pseudopriestleyi* FRX01 MAG was not detected. The *Pseudanabaenaceae cyanobacterium* MP8IB2.15 MAG was also found in a dry sand community in the McMurdo Dry Valleys (37.45% containment), where lakes Vanda and Fryxell are located, as well as Whaler’s Bay on Deception Island in Antarctic (18.31% containment) and the Canadian High Arctic such as Nunavut, Canada (33.54% containment), which is cold but geographically distant from Antarctica.

Metagenomes representing geographically distinct locations were selected for further analysis to compare genomic data from different environments to the Antarctic MAGs. These data sets were run through an assembly and binning pipeline to obtain bins that could be compared to the Antarctic MAGs. However, metagenome assemblies were poor quality with the majority of the N50s under 1,000 base pairs, which is the minimum contig length required to bin with MetaBAT. Thus, bins were not generated likely due to insufficient sequencing depth, and it would not have been possible to identify the presence of the MAGs in these metagenomes without using an assembly-independent technique. Validation of the branchwater results was done by mapping the MAGs to metagenomes (Table 2). The percentage of the MAG detected in metagenome and average MAG coverage confirm the results of branchwater independent of k-mer comparisons, with all but one sample exhibiting higher mapping-based detection in the metagenome than k-mer containment.

## 4 Discussion

### 4.1 Environmental diversity of *Microcoleus* sp. MP8IB2.171

The presence of the *Microcoleus* sp. MP8IB2.171 MAG in diverse environments indicates that it can survive in a range of different ecological conditions and climatic zones. The findings agree with previous biogeographic assessments of cultured cyanobacteria belonging to the species *Microcoleus vaginatus* and the *Microcoleus* spp. based on the 16S rRNA gene (Dvořák et al., 2012; Strunecký et al., 2013). To survive cold temperatures in Lake Vanda, *Microcoleus* sp. MP8IB2.171 must deal with cellular membranes becoming brittle and slowed metabolism. However, some environments where the *Microcoleus* sp. MP8IB2.171 was found are only cold for part of the year (Moab Green Butte Desert; Ningxia, China; Southwest Germany; Milwaukee, Wisconsin; and the United Kingdom) while other environments are cold year-round (Puca Glacier, Peru, and glacial snow in China). In contrast to cold conditions, hot temperatures can cause proteins to denature, and prolonged exposure to sunlight can cause high light and UV stress. These conditions occur in the Moab Green Butte Desert, the Sonoran Desert, and the Negev Desert. Furthermore, the Moab Desert and Sonoran Desert experience extreme temperature changes between morning and night (Turnage and Hinckley, 1938; Balling et al., 1998; McCann et al., 2018), forcing the *Microcoleus* sp. MP8IB2.171 to persist through both conditions on a 24-h cycle.

In addition to temperature range, the *Microcoleus* sp. MP8IB2.171 MAG was found in metagenomes from environments with different levels of water availability and habitat types. Locations included arid desert soil crusts (Moab Desert, USA and Negev Desert, Israel), mine tailings (Shaoyang, China; the United Kingdom; Milwaukee, USA), freshwater rivers (Qing River, China), saline lakes (Ace Lake, Antarctica), and plant microbiomes (wild *Arabidopsis*, Germany). The *Microcoleus* sp. MP8IB2.171 MAG was also found in data from both high and low elevation environments (5,800 m elevation in glacial snow in China and 0 m elevation in the Negev Desert). Overall, the variety of conditions where the *Microcoleus* sp. MP8IB2.171 MAG was found indicates that it may live in an impressive range of environments spanning moderate climates to extreme heat or cold.

### 4.2 Environmental diversity of *Phormidium pseudopriestleyi* FRX01

*P. pseudopriestleyi* FRX01 is a sulfide-tolerant cyanobacteria found in a low light environment in Lake Fryxell, Antarctica. Our study identified the *P. pseudopriestleyi* FRX01 MAG in metagenomes from additional locations in Antarctica such as the saline Ace Lake (Vestfold Hills) and lakes on the Rauer Islands, which agrees with previous 16S rRNA gene sequencing where the species was documented from Salt Pond and Fresh Pond on McMurdo Ice Shelf (Jungblut et al., 2005; Lumian et al., 2021) as well as Ace Lake (Taton et al., 2006). Interestingly, *P. pseudopriestleyi* FRX01 or a close relative is present also at low abundance in a pond at Les Salins du Lion, a bird reserve (20.63% containment, 95% cANI), and Étang de Berre, a hydrocarbon polluted saline lagoon (18.25% containment, 94% cANI), both in southern France (Aubé et al., 2016). Four environmental conditions can be compared in these locations: irradiance, salinity, temperature, and sulfide concentrations. The irradiance at Les Salins du Lion pond and Étang de Berre lagoon was not measured when environmental sampling occurred, but the elevation of the lagoon was recorded to be at 0 m, and we infer that irradiance was higher at the surface of the pond than the low irradiance at the depth of sampling in Lake Fryxell (1–2 μmol/photon m^−2^ s^−1^) (Sumner et al., 2015). Furthermore, Salt Pond and Fresh Pond have high illumination levels in the summer (Roos and Vincent, 1998; Jungblut et al., 2005), indicating that *P. pseudopriestleyi* FRX01 may have the capability to overcome high irradiation and UV fluxes for prolonged periods. Les Salins du Lion (14 g L^−1^ NaCl) and Étang de Berre (20 g L^−1^ NaCl) have a lower salinity than 9.8 m in Lake Fryxell (70.13 g L^−1^ NaCl) and Salt Pond (~990 g L^−1^ NaCl), which is hypersaline (Jungblut et al., 2005; Aubé et al., 2016; Lumian et al., 2021). Previous work has showed that a close relative of *P. pseudopriestleyi* FRX01 (*Oscillatoria acuminata* Jungblut et al., 2016) increases the thickness of its extracellular polymeric substance layer in response to saline stress (Agrawal and Singh, 1999). Sulfide is also present in Les Salins du Lion, with a concentration of ~0.24 g L^−1^ at the time of sampling (Aubé et al., 2016), which was the highest value at any location or time sampled included in the study. This indicates a higher sulfide tolerance than what was previously recorded in the Lake Fryxell sampling site, which was 9.8 × 10–5 g L^−1^ (Lumian et al., 2021).

In addition to Les Salins du Lion and Étang de Berre, *P. pseudopriestleyi* FRX01 MAG genome content was found in globally distributed challenging environments such as a salt flat in Chile, antimicrobial treated sewage in Kenya, and infant gut, where it may be ingested material or contamination. The fact that *P. pseudopriestleyi* FRX01 thrives in environments with harsh conditions suggests that it has capabilities to overcome diverse environmental stresses. In Lake Fryxell, *P. pseudopriestleyi* FRX01 dominates microbial mats at 9.8 m depth in low light and sulfidic conditions but it is less abundant at shallower depths, even though there is more light availability and no sulfide (Jungblut et al., 2016; Dillon et al., 2020). Thus, *P. pseudopriestleyi* FRX01 may grow slowly and find ecological success in environments that are too harsh for faster growing cyanobacteria, which is consistent with the slow growth rate of *P. pseudopriestleyi* FRX01 seen in unpublished laboratory observations. The other environments where genomes similar to *P. pseudopriestleyi* FRX01 were found may provide challenges that prohibit many other cyanobacteria from growing (polar environments, alkaline lake Big Soda Lake, antimicrobial treated sewage in Nairobi, Kenya), allowing *P. pseudopriestleyi* FRX01 to become sufficiently abundant to be represented in metagenomes from nonpolar environments.

### 4.3 Environmental diversity of Pseudanabaenaceae cyanobacterium MP8IB2.15, Leptolyngbyaceae cyanobacterium P9P1.79 and *Leptolyngbya* sp. BulkMat.35

The top matches for the *Pseudanabaenaceae cyanobacterium* MP8IB2.15, *Leptolyngbyaceae cyanobacterium* MP9P1.79, and *Leptolyngbya* sp. BulkMat.35 MAGs showed that they were also present in Lake Fryxell and that the *Pseudanabaenaceae cyanobacterium* MP8IB2.15 MAG was in sediment in the McMurdo Dry Valleys. The *Leptolyngbyaceae cyanobacterium* MP9P1.79 MAG was only present in the McMurdo Dry Valleys, however the presence of the *Leptolyngbya* sp. BulkMat.35 and *Pseudanabaenaceae cyanobacterium* MP8IB2.15 MAGs in geographically distant locations in the Arctic (Norway and Canada respectively) suggests that the cyanobacteria forming these MAGs have a global distribution in cold environments and might have undergone long range dispersal. The mechanism of long-range distribution could be wind; atmospheric studies show bacteria from the Saharan desert are transported by wind throughout the Atlantic (Griffin et al., 2002; Gorbushina et al., 2007; Jungblut et al., 2010). A similar process is expected to allow Antarctic cyanobacteria to cross large distances and populate diverse geographic regions. However, the lack of non-polar locations in metagenomes may suggest that they are not as successful at integrating into non-polar environments. Thus, these cyanobacteria may be specific to polar environments even though they may be transported globally, which agrees with 16S rRNA gene analysis that proposed the presence of cosmopolitan cold ecotypes (Jungblut et al., 2010).

### 4.4 Implications for biogeographic distributions

The perceived distributions of organisms in biogeography studies are affected by sampling and publishing biases. Sampling in remote locations is logistically difficult and is often centered around established sampling locations which may be near research stations and infrastructure. This results in many studies and publications from established sampling locations and a deeper understanding of local ecology and geochemical processes in these environments. Biogeography studies, however, benefit from widespread sampling in many locations. Conducting widespread ecological sampling is expensive and can be impractical, so it is advantageous to search existing datasets for as much information as possible. Using branchwater to search public metagenomes makes the most out of data from remote areas by revealing previously unknown locations of organisms of interest. Furthermore, results from this analysis included remote areas, including various sites in Antarctica, which may not have otherwise been identified as locations of the query MAGs. Finally, the rapid rate of metagenome additions to the SRA database suggests that this technique will become increasingly valuable. For example, the number of metagenomes nearly doubled between construction of our dataset in September 2020 and final revisions in January 2024. Reanalysis would likely identify additional locations for globally distributed organisms whereas it may not for endemic organisms.

Despite being affected by sampling bias like all biogeography studies, the results showed that the *Microcoleus* sp. MP8IB2.171 MAG was globally distributed over a wide variety of environments, the *P. pseudopriestleyi* FRX01 MAG was found in predominantly in harsh environments, the *Leptolyngbyaceae cyanobacterium* MP9P1.79 was only in the Antarctic, and the *Leptolyngbya* sp. BulkMat.35 MAG and the *Pseudanabaenaceae cyanobacterium* MP8IB2.15 MAGs were in geographically separated polar environments. The numerous sites containing the *Microcoleus* sp. MP8IB2.171 MAG imply that this species has the genetic capacity to adapt to many types of environments. It may also have a faster growth rate than an extreme conditions specialist, like *P. pseudopriestleyi* FRX01, which would allow it to compete in a variety of ecological communities, some of which experience stressful conditions. Previous work has shown *Microcoleus sensu stricto* to be a cosmopolitan genus (Garcia-Pichel et al., 1996, 2001).

Although the *Microcoleus* sp. MP8IB2.171 MAG is by far the most globally diverse cyanobacterial genome in this study, there is variety in the distributions of the other four MAGs. The prevalence of the *P. pseudopriestleyi* FRX01 MAG in harsh environments indicates that it finds ecological success in stressful conditions, and it is likely outperformed by other organisms in moderate environments. The *Pseudanabaenaceae cyanobacterium* MP8IB2.15, *Leptolyngbyaceae cyanobacterium* MP9P1.79, and *Leptolyngbya* sp. BulkMat.35 MAGs were only found in polar environments, indicating they may be outcompeted in moderate environments. Diving deeper into the metabolic potential of each organism and interactions between metagenome community members may offer insights as to how and why some organisms are prevalent in a multitude of environments while others are prevalent in only certain conditions.

## 5 Conclusion

This paper presents the first biogeography study using a large-scale k-mer-based approach and characterizes the global distribution of five distinct Antarctic cyanobacteria based on public data. We show that the *Microcoleus* sp. MP8IB2.171 MAG has cosmopolitan distribution and presence in a variety of environments, whereas the *P. pseudopriestleyi* FRX01 MAG is also globally distributed but mostly present in harsh environments. *Leptolyngbya* sp. BulkMat.35, and *Pseudanabaenaceae cyanobacterium* MP8IB2.15 MAGs were only found in polar environments from Arctic to Antarctica suggesting the existence of cosmopolitan cold ecotypes. The *Leptolyngbyaceae cyanobacterium* MP9P1.79 MAG was only detected in Antarctica and provides support for more restricted distribution patterns and potential endemicity. Further *in situ* transcriptomic studies of these MAGs may reveal adaptation mechanisms including why the *Microcoleus* sp. MP8IB2.171 is so pervasive compared to the other cyanobacteria in this study.

Branchwater can search ~500,000 metagenomes with a query genome in under 24 h on commodity hardware (Irber et al., 2022a,b). The ability to quickly find genomes similar to query MAGs in publicly available unassembled metagenomic data sets has important implications for biogeography studies, which have been predominantly based on 16S rRNA gene sequencing due to the prevalence of data and ease of comparison. Branchwater greatly increases the amount of data that can be used for biogeography studies. This technique is especially helpful for organisms that are in remote locations and underrepresented in genomic data, such as polar cyanobacteria, by providing a much larger number of known environments than would be possible with targeted field studies. Additionally, branchwater can be used to identify accessible sampling locations of organisms from remote environments, such as the *Microcoleus* sp. MP8IB2.171 being identified in the Moab Green Butte Desert in Colorado, USA at 41.10% containment. As more metagenome datasets are made publicly available on the NCBI SRA, more information about the distribution of cryosphere cyanobacteria can be attained. The results further demonstrate the potential of metagenomics and k-mer based MAG approaches in investigating biogeography and ecology of cyanobacteria and environmental microbiology in the polar regions.

## Data availability statement

Publicly available datasets were analyzed in this study. This data can be found at: https://www.ncbi.nlm.nih.gov/, SRR6448204-SRR6448219 and SRR6831528, GCF_017313335.1, JARCMA000000000.1, JARCMB000000000.1, JARCMC000000000.1, JARCMD000000000.1.

## Author contributions

JL: Conceptualization, Data curation, Formal analysis, Investigation, Methodology, Software, Visualization, Writing – original draft, Writing – review & editing. DYS: Conceptualization, Funding acquisition, Resources, Supervision, Writing – review & editing. CLG: Conceptualization, Investigation, Validation, Writing – review & editing. ADJ: Methodology, Supervision, Writing – review & editing. LI: Conceptualization, Data curation, Formal analysis, Methodology, Resources, Software, Validation, Writing – review & editing. NT-W: Formal analysis, Investigation, Methodology, Resources, Software, Validation, Writing – review & editing. CT: Conceptualization, Formal analysis, Funding acquisition, Investigation, Methodology, Resources, Software, Supervision, Writing – review & editing.
