# Supplementary material for: Biogeographic distribution of five Antarctic cyanobacteria using large-scale k-mer searching with sourmash branchwater
Source: Front Microbiol. 2024 Feb 19;15:1328083. doi: 10.3389/fmicb.2024.1328083 (PMC10909832; doi:10.3389/fmicb.2024.1328083)
Supplement: Supplementary file 1 [file Data_Sheet_1.PDF]

Supplementary Material

Biogeographic Distribution of Five Antarctic Cyanobacteria Using Large-Scale k-mer Searching with sourmash branchwater

Jessica Lumian, Dawn Sumner, Christen Grettenberger, Anne D. Jungblut, Luiz Irber, Tessa Pierce-Ward, C. Titus Brown*

*** Correspondence:** Corresponding Author: ctbrown@ucdavis.edu

1 Supplementary Tables

Table S1 Metagenomes from the NCBI SRA with the Highest Containment Values

MAG	Containment (%)	Location	Accession Number	Latitude and Longitude	BioSample Metadata[#]
Microcoleus sp. MP8IB2.171	99.18*	Mat lift-off from Lake Fryxell, Antarctica	SRR5468150	77.605 S, 163.1630 E	Isolation Source: Ice surface mat<br><br>Collection Date: 2014-12-05
	65.02*	Polar Desert Sand Communities, Antarctica	SRR6266358	78.0741 S, 163.8918 E	Isolation Source: Antarctic Sand<br><br>Collection Date 2010-01-15
	57.50*	Moab Green Butte, Utah, USA	SRR5855414	38.42 N, 109.41 W	Isolation Source: biocrust samples from Green Butte Site near Canyonlands National Park along an apparent maturity gradient of Cyanobacteria-dominated biocrusts<br><br>Collection Date: 2014-09<br><br>Sample Collection: soil coring with Petri dishes
	41.65*	Ningxia, China	SRR2952554	Not provided	Isolation Source: algae crusts

					Collection Date: 2013-04-15<br><br>Geographic Location: China: Ningxia
	41.10*	Sonoran Desert, Colorado Plateau, USA	SRR5247052	38.42 N, 109.4099 W	Isolation Source: Colorado Plateau and Sonoran Desert<br><br>Plateau and Sonoran Desert
	40.61*	Pig Farm, UK	ERR3588763	55.95 N,<br><br>-3.188 W	Environmental Context: Pig farm soil<br><br>Collection Date: 2017-01-11<br><br>Project Name: The dynamics of antimicrobial resistance gene prevalence on a commercial pig farm: implications for policy
	39.54*	Glacier Snow, China	SRR5891573	38.2186 N, 81.120 E	Environmental Context: Glacier snow from glacier<br><br>Collection Date: 2013-09<br><br>Depth: 0.01 m<br><br>Elevation: 5800 m
	38.36*	Mine Tailing Pool Sediment near Shaoyang, China	ERR1333181	27.745 N, 111.46 E	Environmental Context: Mine tailing pool, sediment<br><br>Collection Date: 2014-12-27<br><br>Depth: 0.1 m (?)<br><br>Elevation: 286 m (?)
	37.04*	Wastewater in Milwaukee, Wisconsin, USA	SRR5459769	43.023 N, 87.895 W	Environmental Context: Wastewater communities<br><br>Collection Date: 2014-07-17

	36.30*	Puca Glacier, Peru	SRR6048908	13.773 S, 71.071 W	Environmental Context: Early successional soil, N + P addition<br><br>Collection Date: 2012
	35.71*	Negev Desert, Israel	SRR12473531	30.785 N, 34.767 E	Environmental Context: Temperate “Mediterranean” desert biome<br><br>Collection Date: 2017-05-10<br><br>Depth: 0.2 cm<br><br>Elevation: 0 m
	33.58*	Southwest Germany	ERR3192241	Not Provided	BioProject Information: Short read whole genome sequencing of 276 wild *Arabidopsis thaliana* rosettes from southwest Germany
P. pseudopriestleyi FRX01	98.49*	Microbial mat in Lake Fryxell, Antarctica	SRR7769747	77.6167 S, 163.1833 E	Collection Date: 2012-11<br><br>Isolation source: benthic surface of ice-covered lake
	55.80*	Ace Lake, Antarctica	SRR7528444	68.473 S, 78.188 E	Collection Date: 2014-02-15<br><br>Isolation Source: saline lake
	23.54*	Rauer Islands, Antarctica	SRR5216658	68.556 S, 78.191 E	Collection Date: 2015-01-11<br><br>Isolation Source: saline lake
	20.63*	Les Salins du Lion Bird Reserve, France	SRR7428116	43.453 N, 5.230 E	Environmental Context: Microbial mat from brackish lagoon in a natural zone of ecological interest<br><br>Collection Date: 2011-09<br><br>Depth: 0.2 cm<br><br>Elevation: 0 m

	19.04*	Big Soda Lake, Nevada	SRR12522841	39.523 N, 118.870 W	Collection Date: 2016-06-22
	18.25*	Étang de Berre Lagoon, France	SRR7428132	43.485 N, 5.188 E	Environmental Context: Microbial mat from hydrocarbon retention basic<br><br>Collection Date: 2012-04<br><br>Depth: 0.2 cm<br><br>Elevation: 2 m
	11.99*	Sewage in Nairobi, Kenya	ERR3503286	1.19 S, 36.47 E	Environmental Context: Stream collection for survey of infectious diseases and antimicrobial resistance<br><br>Collection Date: 2014-07-28
	10.37	Wetland soil in Yanghu, China	SRR9691033	29.19 N, 90.59 E	Collection Date: 2013-07-12
	8.98*	Salar del Huasco salt flat, Chile	SRR10186387	20.264 S, 68.875 W	Collection Date: 2018-06-02
	8.48*	Simulated Metagenome	ERR738546	Not Applicable	Simulated metagenome based on real Illumina HiSeq 2000 data to benchmark metagenome analysis tools
	7.61*	Human Gut	SRR6262267	40.431 N, 79.959 W	Sample Context: Fecal sample from infant<br><br>Collection Date: 2015
Pseudanabaenaceae cyanobacterium MP8IB2.15	99.49*	Mat lift-off from Lake Fryxell, Antarctica	SRR5468149	78.072 S, 163.719 E	Collection Date: 2009-01-15<br><br>Isolation Source: Antarctic Sand

	37.45	Dry Valley Sand Communities , Antarctica	SRR6266338	81.017 N, 81.583 W	Collection Date: 2015-07-18<br><br>Organism: Glacier metagenome
	33.54*	Nunavut, Canada	SRR5829599	77.605 S, 163.1630 E	Isolation Source: Ice surface mat<br><br>Collection Date: 2014-12-05
	18.31*	Deception Island, Antarctica (Whaler's Bay Sediment)	ERR4192538	62.97 S, 60.55 W	Collection Date: 2017-12<br><br>Broad Scale Environmental Context: Lake<br><br>Local Scale Environmental Context: Lake<br><br>Environmental Medium: sediment
	7.45*	Microbial mat in Lake Fryxell, Antarctica	SRR7769784	77.6167 S, 163.1833 E	Collection Date: 2012-11<br><br>Isolation Source: Benthic surface of ice-covered lake<br><br>Host: Lake<br><br>pH: 7.47
Leptolyngbyaceae cyanobacterium MP9P1.79	97.82*	Mat lift-off from Lake Fryxell, Antarctica	SRR5208701	77.605 S, 163.163 E	Collection Date: 2014-12-05<br><br>Isolation source: Ice surface mat
Leptolyngbya sp. BulkMat.35	98.72*	Mat lift-off from Lake Fryxell, Antarctica	SRR5468150	77.605 S, 163.163 E	Collection Date: 2014-12-05<br><br>Isolation Source: Ice surface mat
	8.40	Spitsbergen, Svalbard, Norway, Arctic	SRR6683740	78.58 N, 12.05 E	Collection Date: 2016-07-01<br><br>Isolation Source: Soil and sediment

An asterisk (*) denotes where multiple samples from the same location above 5% containment were identified but are not shown in this table, which only shows distinct geographical hits. Only the

sample with the highest containment is shown. For an extended full list of hits, see Supplementary Tables 4 – 9.

Note: Question marks are used where units are assumed but not given in SRA metadata. #Metadata provided as available from SRA, which differed for many datasets.

Table S2 Additional Metagenomes from Unique Locations >20% Containment for *Microcoleus* MAG

MAG	Containment (%)	Location	Latitude and Longitude	Accession Number
Microcoleus sp. MP8IB2.171	32.14	Qing River, China	40.029 N, 116.368 E	SRR10571243
	31.77	Fecal Metagenome of Great Black-Headed Gulls around Qinghai Lake, China	36.78 N, 100.00 E	SRR10492798
	31.35	Agave Microbial Communities from Guanajuato, Mexico	21.766 N, 100.163 W	SRR4142282
	31.14	Miers Valley, Antarctica	78.160 S, 164.100 E	SRR3471615
	31.06	Glacial meltwater from Laohugou glacier, China	39.50 N, 96.52 E	SRR9965273
	28.87	Polar Desert Sand Communities, Antarctica	78.024 S, 163.917 E	SRR6266336
	28.77	Ace Lake, Antarctica	68.473 S, 78.188 E	SRR7528444
	28.41	Particulate Aerosol Particulate Matter, Beijing, China	40.01 N, 116.33 E	SRR10613504
	28.40	Soil crust, Chicken Creek, Germany	51.36 N, 14.15 E	SRR8357461
	28.17	Wetland soil, Lanzhou, China	36.09 N, 103.71 E	SRR9691044
	27.50	Terrestrial metagenome from Alberta, Canada	50.34 N, 113.77 W	SRR5678923

	27.15	Cave, Twin Sisters, Idaho	42.02 N, 113.72 W	SRR7774479
	25.88	Soil communities, Rifle, Colorado	39.53 N, 107.78 W	SRR3969602
	25.27	Semi-synthetic marine metagenomes from University of Algarve, Faro, Portugal	NA	ERR1992808
	25.25	Saskatchewan, Canada	50.28 N, 107.80 W	SRR7013884
	24.64	Wheat and chickpea soil microbiome, Australia	34.538 S, 138.690 E	ERR3029103
	23.58	Soil communities, Uluru, Australia	25.350 S, 131.052	ERR671932
	22.19	Rhizosphere soil, Mafikeng, South Africa	25.79 S, 25.61 E	SRR11128415
	20.79	Microbial mat, Eel River, California, USA	39.840 N, 123.710 W	SRR244337
	20.33	Biofilm in Wai-iti River, New Zealand	NA	SRR9948934
	20.29	Soil rhizosphere, Durango, Mexico	19.321 N, 99,194 W	SRR11092592

Table S3 Quality Metrics of Metagenome Assemblies and Mapping Statistics

MAG	SRA Accession Number and Location	Number of Contigs (over 500 bp)	Total Length of Contigs (bp, over 500 bp)	Largest Contig (bp)	N50
Microcoleus sp. MP8IB2.171	SRR5855414 Moab Green Butte, Utah, USA	779, 743	497,286,103	4,248	612
	SRR2952554 Ningxia, China	296,291	187,141,964	7,947	604

	SRR5247052 Sonoran Desert, Colorado Plateau, USA	781,528	498,867,695	3,982	613
	ERR3588763 Pig Farm, UK	402,432	243,270,443	3,395	581
	SRR5891573<br><br>Glacier Snow, China	979,446	619,490,337	3,614	606
	ERR1333181 Antimony Polluted Sediment, China	415,411	260,914,760	5,701	607
	SRR5459769<br><br>Wastewater in Milwaukee, Wisconsin USA	1,259,786	1,086,536,868	57,883	819
	SRR12473531<br><br>Negev Desert, Israel	297,517	187,591,328	4,696	600
	ERR192241<br><br>Southwest Germany	108,078	63,950,717	1,868	567
P. pseudopriestleyi FRX01	SRR7528444<br><br>Ace Lake, Antarctica	218,966	195,047,488	62,020	822
	SRR5216658<br><br>Rauer Islands, Antarctica	250,605	212,798,142	64,599	794
	SRR7428116<br><br>Bird Reserve, France	462,930	371,142,339	36,453	146,664
Pseudanabaenaceae cyanobacterium MP8IB2.15	SRR6266338<br><br>Dry Valley, Antarctica	651,239	424,874,538	9,934	624
	SRR5829599 Canada	704,963	564,862,171	27,496	751
Leptolyngbyaceae cyanobacterium MP9P1.79	SRR5468153 Lake Fryxell, Antarctica	1,119,433	887,670,078	21,258	760

Leptolyngbya sp. BulkMat.35	SRR5468153 Lake Fryxell, Antarctica	1,119,433	887,670,078	21,258	760

Supplemental Table S4 Extended Matches from branchwater Leptolyngbyaceae cyanobacterium MP9P1.79 MAG Hits >5% Containment

MATCHES	CONTAINMENT	LOCATION
SRR5208701	97.82%	Lake Fryxell liftoff and glacier meltwater
SRR5468149	97.16%	Lake Fryxell liftoff and glacier meltwater
SRR5208700	81.58%	Lake Fryxell liftoff and glacier meltwater
SRR5208699	74.52%	Lake Fryxell liftoff and glacier meltwater
SRR5468150	65.46%	Lake Fryxell liftoff and glacier meltwater
SRR5468153	36.51%	Lake Fryxell liftoff and glacier meltwater

Supplemental Table S5 Extended Matches from branchwater *Leptolyngbya* sp. BulkMat.35 MAG Hits >5% Containment

MATCHES	CONTAINMENT	LOCATION
SRR5468150	98.72%	Lake Fryxell liftoff and glacier meltwater
SRR5468149	98.58%	Lake Fryxell liftoff and glacier meltwater
SRR5208699	98.35%	Lake Fryxell liftoff and glacier meltwater
SRR5208701	97.98%	Lake Fryxell liftoff and glacier meltwater
SRR5208700	97.70%	Lake Fryxell liftoff and glacier meltwater
SRR5468153	25.16%	Lake Fryxell liftoff and glacier meltwater
SRR6683740	8.40%	Arctic Lake Metagenome

Supplemental Table S6 Extended Matches from branchwater Pseudanabaenaceae cyanobacterium MP8IB2.15 MAG Hits >5% Containment

MATCHES	CONTAINMENT	LOCATION
SRR5468149	99.49%	Lake Fryxell liftoff and glacier meltwater
SRR5468150	99.46%	Lake Fryxell liftoff and glacier meltwater
SRR5208701	99.20%	Lake Fryxell liftoff and glacier meltwater
SRR5468153	99.13%	Lake Fryxell liftoff and glacier meltwater
SRR5208700	98.91%	Lake Fryxell liftoff and glacier meltwater
SRR5208699	98.12%	Lake Fryxell liftoff and glacier meltwater
SRR6266338	37.45%	Polar Desert Sand Communities
SRR5829599	33.54%	Nunavut, Canada
SRR5215118	30.57%	Nunavut, Canada
SRR5829597	26.16%	Nunavut, Canada
ERR4192538	18.31%	Deception Island, Antarctica (Whaler's Bay Sediment)
ERR4192539	16.43%	Deception Island, Antarctica (Whaler's Bay Sediment)
SRR7769784	7.45%	Antarctic Microbial Mat
SRR7769706	6.84%	Antarctic Microbial Mat
SRR7769810	5.46%	Antarctic Microbial Mat
SRR8842248	5.39%	Cryoconite from Svalbard
SRR7769748	5.21%	Antarctic Microbial Mat

Supplemental Table S7 Extended Matches from branchwater *Phormidium pseudopriestleyi* FRX01 MAG Hits >5% Containment

MATCHES	CONTAINMENT	LOCATION
SRR7769578	99.39%	Antarctic Microbial Mat
SRR7769621	99.32%	Antarctic Microbial Mat
SRR7769581	98.49%	Antarctic Microbial Mat
SRR7769747	98.49%	Antarctic Microbial Mat
SRR7769746	98.44%	Antarctic Microbial Mat
SRR7769635	98.25%	Antarctic Microbial Mat
SRR7769622	98.18%	Antarctic Microbial Mat
SRR7769634	98.11%	Antarctic Microbial Mat
SRR7769582	98.07%	Antarctic Microbial Mat
SRR7769583	97.79%	Antarctic Microbial Mat
SRR7769576	97.74%	Antarctic Microbial Mat
SRR7769579	97.60%	Antarctic Microbial Mat
SRR7769748	97.15%	Antarctic Microbial Mat
SRR7769793	96.56%	Antarctic Microbial Mat
SRR7769792	95.86%	Antarctic Microbial Mat
SRR7769639	94.70%	Antarctic Microbial Mat
SRR7769754	93.97%	Antarctic Microbial Mat
SRR7769518	92.65%	Antarctic Microbial Mat
SRR7769683	92.01%	Antarctic Microbial Mat

SRR7769753	91.90%	Antarctic Microbial Mat
SRR7769616	91.83%	Antarctic Microbial Mat
SRR7769558	91.00%	Antarctic Microbial Mat
SRR7769636	90.18%	Antarctic Microbial Mat
SRR7769554	88.88%	Antarctic Microbial Mat
SRR7769620	85.02%	Antarctic Microbial Mat
SRR7769624	83.72%	Antarctic Microbial Mat
SRR7769514	82.76%	Antarctic Microbial Mat
SRR7769557	81.55%	Antarctic Microbial Mat
SRR7769751	80.54%	Antarctic Microbial Mat
SRR7769794	77.45%	Antarctic Microbial Mat
SRR7769755	61.13%	Antarctic Microbial Mat
SRR7528444	55.79%	Ace Lake Saline
SRR7769788	54.53%	Antarctic Microbial Mat
SRR7529760	47.77%	Ace Lake Saline
SRR7769790	42.68%	Antarctic Microbial Mat
SRR7769643	39.13%	Antarctic Microbial Mat
SRR7769519	29.52%	Antarctic Microbial Mat
SRR7769812	25.39%	Antarctic Microbial Mat
SRR5216658	23.54%	Rauer Islands, Antarctica (saline)
SRR7769513	22.92%	Antarctic Microbial Mat

SRR6129595	22.38%	Rauer Islands, Antarctica (saline)
SRR7769618	22.10%	Antarctic Microbial Mat
SRR6185695	21.16%	Rauer Islands, Antarctica (saline)
SRR7529754	20.83%	Ace Lake Saline
SRR7428116	20.63%	Brackish Lagoon (SL)
SRR7428117	20.33%	Brackish Lagoon (SL)
SRR7428114	20.28%	Brackish Lagoon (SL)
SRR7769787	19.27%	Antarctic Microbial Mat
SRR7428121	19.17%	Brackish Lagoon (SL)
SRR7428120	19.10%	Brackish Lagoon (SL)
SRR12522841	19.04%	Big Soda Lake, Nevada
SRR7428115	18.87%	Brackish Lagoon (SL)
SRR7769528	18.47%	Antarctic Microbial Mat
SRR7428132	18.25%	Brackish Lagoon (EBD)
SRR12522839	17.71%	Big Soda Lake, Nevada
SRR7769662	16.79%	Antarctic Microbial Mat
SRR7769657	16.75%	Antarctic Microbial Mat
SRR7769512	16.66%	Antarctic Microbial Mat
SRR12522840	16.52%	Big Soda Lake, Nevada
SRR7769693	16.11%	Antarctic Microbial Mat
SRR7529732	15.95%	Ace Lake Saline
SRR7769584	15.20%	Antarctic Microbial Mat

SRR7428131	14.98%	Brackish Lagoon (EBD)
SRR7769531	14.14%	Antarctic Microbial Mat
SRR7769802	14.11%	Antarctic Microbial Mat
SRR7769623	13.08%	Antarctic Microbial Mat
SRR7769574	13.01%	Antarctic Microbial Mat
SRR7769801	12.98%	Antarctic Microbial Mat
SRR7769678	12.70%	Antarctic Microbial Mat
SRR7769659	12.16%	Antarctic Microbial Mat
ERR3503286	11.99%	Nairobi, Kenya (sewage, antimicrobial resistance)
SRR7769811	11.76%	Antarctic Microbial Mat
SRR7529753	11.73%	Ace Lake Saline
SRR7769810	11.28%	Antarctic Microbial Mat
SRR7769559	11.26%	Antarctic Microbial Mat
SRR7769735	11.14%	Antarctic Microbial Mat
SRR7769804	10.96%	Antarctic Microbial Mat
SRR7769696	10.95%	Antarctic Microbial Mat
SRR7769515	10.89%	Antarctic Microbial Mat
SRR7769776	10.50%	Antarctic Microbial Mat
SRR9691033	10.37%	Yanghu, China (wetland soil)
SRR7769805	10.17%	Antarctic Microbial Mat
SRR7769660	10.11%	Antarctic Microbial Mat

SRR7769650	10.03%	Antarctic Microbial Mat
SRR7769752	10.03%	Antarctic Microbial Mat
SRR7769808	10.01%	Antarctic Microbial Mat
SRR7769734	9.47%	Antarctic Microbial Mat
SRR7769773	9.42%	Antarctic Microbial Mat
SRR7769575	9.07%	Antarctic Microbial Mat
SRR10186387	8.98%	Salar de Huasco, Chile (sediment)
ERR3503282	8.83%	Nairobi, Kenya (sewage, antimicrobial resistance)
SRR7769615	8.69%	Antarctic Microbial Mat
SRR7769580	8.64%	Antarctic Microbial Mat
SRR7769695	8.62%	Antarctic Microbial Mat
SRR7769775	8.51%	Antarctic Microbial Mat
SRR12522836	8.48%	Big Soda Lake, Nevada
ERR738546	8.48%	Simulated Metagenome
SRR7769553	8.41%	Antarctic Microbial Mat
SRR7769642	8.36%	Antarctic Microbial Mat
ERR738544	8.10%	Simulated Metagenome
ERR738545	7.87%	Simulated Metagenome
SRR7769664	7.87%	Antarctic Microbial Mat
SRR6262267	7.61%	Human Gut
SRR7428125	7.30%	Brackish Lagoon (EBD)
SRR7769736	7.05%	Antarctic Microbial Mat

SRR7769570	6.95%	Antarctic Microbial Mat
SRR7769803	6.48%	Antarctic Microbial Mat
SRR11412982	6.31%	Human Gut

Supplemental Table S8 Extended Matches from branchwater *Microcoleus* sp. MP8IB2.171 MAG Hits >30% Containment

MATCHES	CONTAINMENT	LOCATION
SRR5468150	99.18%	Lake Fryxell liftoff and glacier meltwater
SRR5468153	99.18%	Lake Fryxell liftoff and glacier meltwater
SRR5208700	98.66%	Lake Fryxell liftoff and glacier meltwater
SRR5468149	98.58%	Lake Fryxell liftoff and glacier meltwater
SRR5208699	86.19%	Lake Fryxell liftoff and glacier meltwater
SRR5208701	84.99%	Lake Fryxell liftoff and glacier meltwater
SRR6266358	65.02%	Polar Desert Sand Communities
SRR5855414	57.50%	Moab Soil Crust
SRR5855413	54.76%	Moab Soil Crust
SRR5855418	53.50%	Moab Soil Crust
SRR5855417	52.39%	Moab Soil Crust
SRR5855428	52.34%	Moab Soil Crust
SRR5855424	48.82%	Moab Soil Crust
SRR5855412	47.99%	Moab Soil Crust
SRR5855429	43.55%	Moab Soil Crust

SRR5855432	42.39%	Moab Soil Crust
SRR2952554	41.65%	Ningxia, China (soil crust)
SRR2954705	41.24%	Ningxia, China (soil crust)
SRR5247052	41.10%	Sonoran Desert
ERR3588763	40.61%	UK Pig Farm
SRR5855420	40.52%	Moab Soil Crust
SRR3439671	40.10%	Ningxia, China (soil crust)
SRR5830676	39.54%	Polar Desert Sand Communities
SRR5891573	39.54%	Glacial Snow, China
ERR1333181	38.36%	Mine Tailing Pool in China
SRR5459769	37.04%	Wastewater, Wisconsin
SRR6048908	36.30%	Puca Glacier, Peru
SRR12473531	35.71%	Mediterranean Desert Community
SRR12473532	35.68%	Mediterranean Desert Community
SRR5855438	34.78%	Moab Soil Crust
SRR12473534	34.30%	Mediterranean Desert Community
ERR3192241	33.57%	Southwest Germany (Arabidopsis community)
